# Neurology practice and stroke services across East China: a multi-site, county-level hospital-based survey

**DOI:** 10.1186/s12883-019-1518-9

**Published:** 2019-11-19

**Authors:** Jun-Fang Zhang, Meng-Yao Qiu, Yu-Lei Zhang, Xi-Xi Wang, Guo-Ping Wang, Yu Geng, Ke-Zhong Zhang, Kan Fang, Yun-Cheng Wu

**Affiliations:** 1 Department of Neurology, Shanghai General Hospital, Shanghai Jiao Tong University School of Medicine, No. 86, Wujin Road, Shanghai, 200080 People’s Republic of China; 2 Department of Neurology, The First Affiliated Hospital of USTC, Division of Life Sciences and Medicine, University of Science and Technology of China, Hefei, Anhui People’s Republic of China; 3 Department of Neurology, Zhejiang Provincial People’s Hospital, People’s Hospital of Hangzhou Medical College, Hangzhou, People’s Republic of China; 4 Department of Neurology, the First Affiliated Hospital of Nanjing Medical University, Nanjing, People’s Republic of China

**Keywords:** Neurology, Stroke center, Hospital services, Survey, Intravenous thrombolysis, Arterial thrombectomy

## Abstract

**Background:**

Neurological disorders are an economic and public health burden which requires efficient and adequate medical resources. Currently, little is known about the status of the quality of neurological care services available in China. As neurological primary care is mostly provided at the county hospital level, investigation of this geographical level is required. The aim of this study is to evaluate currently available neurology care services in Yangtze River Delta Urban Agglomerations in east China.

**Methods:**

A multi-site, county-level hospital-based observational survey was conducted in east China from January 2017 to December 2017. A questionnaire was made to assess hospital and the departmental patient care capabilities, human resources and technical capacity in neurology departments.

**Results:**

Of 228 hospitals across the Yangtze River Delta Urban Agglomerations, 217 documents were returned. Of these, 22 were excluded due to invalid hospital information or duplicate submission. Overall, most hospitals have neurology departments (162, 83.1%) while less than half of the hospitals have a stroke center (80, 41.0%) and neurology emergency department (46, 23.6%). Among 162 hospitals with neurology department, 5 were excluded due to inadequate sharing, leaving 157 hospitals for analysis. About 84.1% of these neurology departments can administer intravenous thrombolysis while about one third of them has the ability to perform arterial thrombectomy (36.9%). In addition, 46.2% of hospitals can carry out computed tomography angiography (CTA) in emergency room. Tertiary care hospitals are much more equipped with modern medical resources compared to the secondary hospitals. In four administrative regions, the neurology services are better in more economically advanced regions.

**Conclusions:**

Neurological care services need to be enhanced at the county-level hospitals to improve health care delivery.

## Background

Neurological disorders are a great threat to public health [1]. The most common neurological disorders, including stroke, Parkinson’s disease, Alzheimer’s disease, epilepsy, headache disorders, multiple sclerosis, neurological infections and traumatic brain injuries, are a significant worldwide economic and health burden. Among those neurological disorders, stroke is the second leading cause of the death in the world and the leading cause of the death in China [2, 3]. According to the Healthcare Access and Quality (HAQ) index, stroke is ranked as the second lowest among 32 diseases or conditions from which death is preventable in China, which suggests that the medical resource spending on stroke care are still insufficient compared with other diseases [4]. To date, research on the availability of neurological care, especially on stroke care in hospital settings has been largely limited to in high income areas such as European countries [5]. There is a paucity of studies investigating the delivery of neurological care services in China, especially at county hospital level which provides neurology primary care services in most areas. One study indicates that the proportion of stroke centers is relatively high in eastern and southern China, and low in northeast and western China [6].

This study evaluates the current status of available neurological care services including hospital and neurological departmental setup, human resources and technical capacity at the county level in the Yangtze River Delta Urban Agglomerations. The Yangtze River Delta Urban Agglomerations has about 150 million people and is one of the most economical active areas in China. With a comprehensive understanding of current status of neurological primary care services in China, more targeted strategies could be undertaken to improve health care, particularly in neurology services in China.

## Methods

### Data collection

**Fig. 1:**
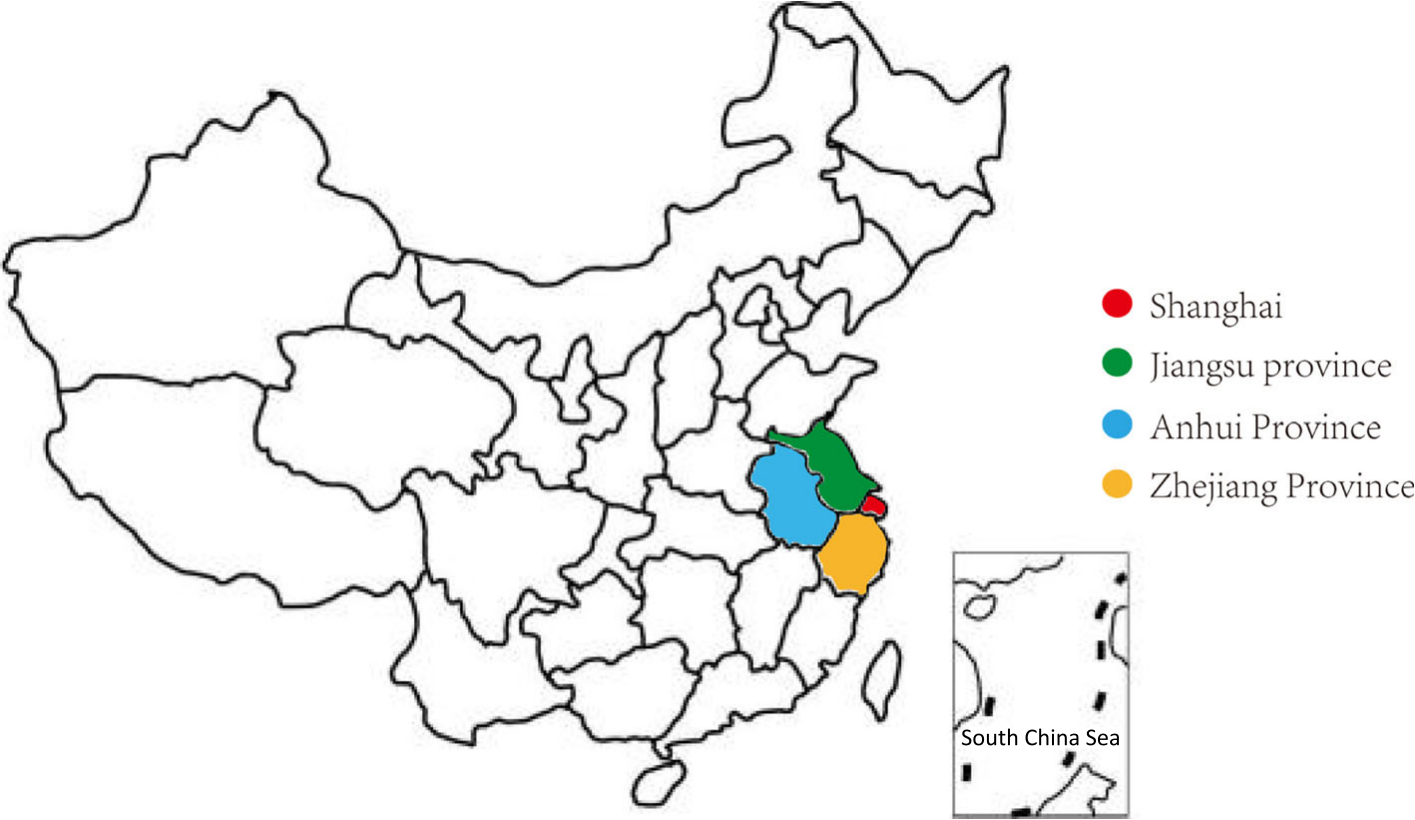
The geographical locations of participating hospitals in Yangtze River Delta Urban Agglomerations of China

This descriptive study was conducted in 228 county-level hospitals from January 2017 to December 2017 in Yangtze River Delta Urban Agglomerations which includes 4 administrative regions of China: Anhui Province, Jiangsu Province, Zhejiang Province and Shanghai **(**Fig. 1**)**. There are totally 34 provincial-level administrative regions (including 4 municipalities directly under the Central Government) in China and we studied four of them. The Yangtze River Delta Urban Agglomerations represents the advanced economically or medically advanced regions. County level is at the third level of the administrative hierarchy in the Chinese hospital system. The first level is the provincial capital hospital (such as comprehensive medical centers at Nanjing in Jiangsu Province, Hangzhou in Zhejiang province, Hefei in Anhui Province, or most university hospital in Shanghai or Beijing), the second level are the comprehensive medical centers in prefecture-level cities (such as municipal hospital in Suzhou, Wuxi, etc.), and the third level are county level hospitals (such as central hospital in each county) or district central hospitals in big cities (discussed here as county level hospital), and the fourth level are community healthcare centers which provide primary medical care.

The investigators contacted the heads of all of the neurology departments or the leaders of the neurology subspecialty in Internal Medicine in county hospitals either by face to face discussion, e-mails or telephone calls, asking them to participate in the study. Once the neurology departments head or the leaders of the neurology subspecialty agreed to participate in the survey, he or she was asked to complete the survey documents and return them by mail. The survey on neurological practice was designed by the committee members of Union of Neurology specialties of Yangtze River Delta Urban Agglomerations (UN-YRDUA).

### Standard protocol approvals

If the participants chose to complete the survey, consent with participants was presumed.

### Survey

Each responding neurology department head (or the leader of neurology subspecialty) received questionnaire documents by either e-mail or post. The cover letter introduced the purpose of the survey, which was to investigate neurology departments and enhance the neurology networks in Yangtze River Delta Urban Agglomerations. The document aimed to assess the hospital and department setup: information about the hospital (hospital level, teaching hospital or not, presence of a stroke center, if yes, comprehensive stroke center or primary stroke center, presence of an independent Neurology Emergency, ability to perform multimodel CTA in emergency departments), information about the department (numbers of neurologists and nurses and their education background, department technologies and department settings).

### Statistical analysis

We performed all analyses with IBM SPSS Statistics for Windows software, version 24.0 (IBM Corp, Armonk, NY). Findings from the analysis were reported in the forms of numbers and percentages displayed in tables underlying the availability of neurological services. Analysis also highlighted variances in available neurological services across the study hospitals.

## Results

**Fig. 2:**
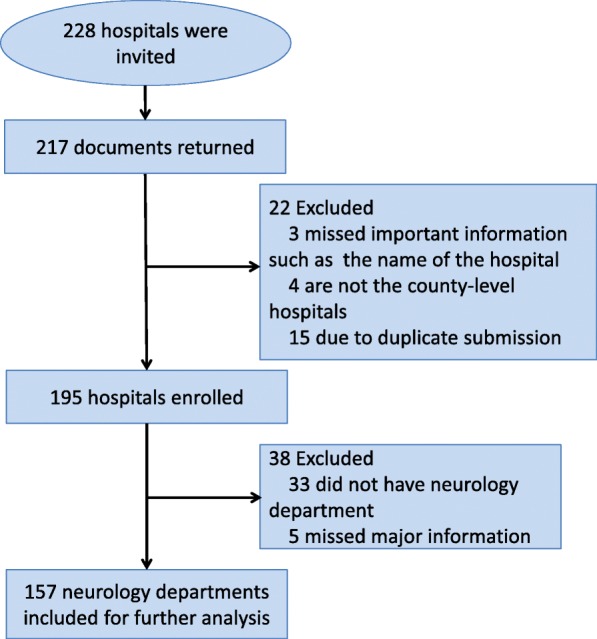
Flow of hospitals through survey

From January 2017 to December 2017, of 228 invited hospitals, a total of 217 documents were returned. Of those 217 hospitals, 22 hospitals were excluded because of inadequate data (3), were designated a non-county level hospital (4) or were duplicate submissions (15). The flow chart is showed in Fig. 2.

### Characteristics

Table 1 summarizes the characteristics of 195 hospitals. Half of them are teaching hospitals (50.8%). Most hospitals are secondary hospitals (67.2%). In 80 hospitals (41.0%) that have stroke centers, 13 (16.3%) of them have comprehensive stroke centers. Less than half (46.2%) of hospitals can carry out CTA in the Emergency Department. Most hospitals (83.1%) have neurology departments while few hospitals have emergency departments for neurology (23.6%). Further analysis was done according to hospital levels and administrative regions (Table 1).

**Table 1:** Characteristics of the 210 participating hospitals according to hospital level and administrative region

Characteristics	Overall (*n* = 195)	Hospital level (*n* = 192)		Administrative region (*n* = 195)			
		Tertiary(*n* = 61)	Secondary(*n* = 131)	Anhui (*n* = 53)	Zhejiang (*n* = 74)	Jiangsu (*n* = 52)	Shanghai(*n* = 16)
Hospital level ^a^							
Tertiary (high level)	61 (31.3%)	–	–	4 (7.5%)	27 (36.5%)	23 (44.2%)	7 (43.8%)
Secondary (moderate level)	131 (67.2%)	–	–	48 (90.6%)	45 (60.8%)	29 (55.8%)	9 (56.3%)
Primary (low level)	0 (0%)	–	–	0 (0%)	0 (0%)	0 (0%)	0 (0%)
Missing ^b^	3 (2.5%)	–	–	1 (1.9%)	2 (2.7%)	0 (0%)	0 (0%)
Teaching hospital							
Yes	99 (50.8%)	52 (85.2%)	47 (35.9%)	15 (28.3%)	34 (45.9%)	39 (75.0%)	11 (68.8%)
No	96 (49.2%)	9 (14.8%)	84 (64.1%)	38 (71.7%)	40 (54.1%)	13 (25.0%)	5 (31.3%)
Stroke center							
Yes	80 (41.0%)	39 (63.9%)	41 (31.3%)	4 (7.5%)	31 (41.9%)	36 (69.2%)	9 (56.3%)
Comprehensive stroke center	13 (16.3%)	8 (20.5%)	5 (12.2%)	0 (0%)	3 (9.7%)	5 (13.9%)	5 (55.6%)
Primary stroke center	67 (83.8%)	31 (79.5%)	36 (87.8%)	4 (100%)	28 (90.3%)	31 (86.1%)	4 (44.4%)
Under construction	64 (32.8%)	18 (29.5%)	46 (35.1%)	24 (45.3%)	28 (37.8%)	9 (17.3%)	3 (18.8%)
No	51 (26.1%)	4 (6.6%)	44 (33.6%)	25 (47.2%)	15 (20.3%)	7 (13.5%)	4 (25.0%)
Multimodel CTA in Emergency Room							
Yes	90 (46.2%)	45 (73.8%)	45 (34.4%)	16 (30.2%)	29 (39.2%)	33 (63.4%)	12 (75.0%)
No	105 (53.8%)	16 (26.2%)	86 (65.6%)	37 (69.8%)	45 (60.8%)	19 (36.5%)	4 (25.0%)
Neurology department							
Yes	162 (83.1%)	61 (100%)	101 (77.0%)	39 (73.6%)	56 (75.7%)	52 (100%)	15 (93.8%)
No	33 (16.9%)	0 (0%)	30 (22.9%)	14 (26.4%)	18 (24.3%)	0 (0%)	1 (6.3%)
Neurology Emergency							
Yes	46 (23.6%)	25 (42.2%)	21 (16.0%)	11 (20.8%)	11 (14.8%)	14 (26.9%)	10 (62.5%)
No	149 (76.4%)	36 (57.8%)	110 (84.0%)	42 (79.2%)	63 (85.1%)	38 (73.1%)	6 (37.5%)

Values are number (percent) when appropriate. *CTA* Computed tomography angiography

^a^ In China, all hospitals are classified into 3 levels: primary, secondary, and tertiary. Primary level hospitals (low level, 100 beds) aim to provide basic public health services and consulting for their residents. Secondary (moderate level, 101–500 beds) and tertiary (high level, > 500 beds) hospitals provide specialized care

^b^ The number of head of neurology departments who finished documentbut did not provide an answer to this item

Of the 162 hospitals that have neurology departments, 5 hospitals were excluded for further analyses because of inadequate information.

After further analyzing the features of 157 neurology departments, characteristics are shown in Table 2. In China, medical students can practice in the hospital after they graduated with bachelor’s degree of medicine and received necessary postdoctoral training. Less than one quarter (22.3%) of neurology departments have physicians who have doctoral degree, while 77.7% of departments have physicians who have a master’s degree. For the stroke services, most hospitals (84.1%) can carry out intravenous thrombolysis while one-third (36.9%) of hospitals can carry out arterial thrombectomy. About half of hospitals (58.6%) have digital subtraction angiography (DSA) technology, but few departments have the ability to carry out complicated procedures such as intracranial arterial stenosis stenting (26.8%), extracranial arterial stenosis stenting (27.4%), intracranial balloon dilatation (15.9%), and extracranial balloon dilatation (14.6%). Few departments can treat the patients with deep brain stimulation (9.6%), transcranial magnetic stimulation (7.6%), botulinum toxin therapy (19.7%), or perform muscle biopsy (14.6%). More than half of the departments can carry out examinations such as electroencephalogram (82.2%), electromyography (65.6%) and transcranial Doppler (56.1%), and 76 (48.4%) hospitals can carry out polysomnography examinations. As for the laboratory test, about 26.1% of hospitals have the drug metabolism genetic testing (such as CYP2C19) and 56.1% have drug concentration testing (such as valproate and carbamazepine). Finally, about one third of hospitals (31.8%) have oligoclonal bands testing. Similarly, further analysis was done according to hospital levels and administrative regions (Table 1).

**Table 2:** Characteristics of the 169 neurology departments hospitals according to hospital level and administrative region

Characteristics	Overall (*n* = 157)	Hospital level (*n* = 157)		Administrative region (*n* = 157)			
		Tertiary (*n* = 61)	Secondary (*n* = 96)	Anhui (*n* = 38)	Zhejiang(*n* = 56)	Jiangsu(*n* = 49)	Shanghai (*n* = 14)
Highest academic degree obtained							
Doctor’s degree ^a^	35 (22.3%)	22 (36.1%)	13 (13.5%)	0 (0%)	6 (10.7%)	16 (32.7%)	13 (92.9%)
Master’s degree	122 (77.7%)	60 (98.4%)	57 (59.4%)	18 (47.4%)	39 (69.6%)	46 (93.9%)	14 (100%)
The availability of neurological care services							
Intravenous thrombolysis	132 (84.1%)	58 (95.1%)	74 (77.1%)	25 (65.8%)	51 (91.1%)	44 (89.8%)	12 (85.7%)
Digital subtraction angiography (DSA)	92 (58.6%)	51 (83.6%)	41 (42.7%)	15 (39.5%)	31 (55.4%)	36 (73.5%)	10 (71.4%)
Arterial thrombectomy	58 (36.9%)	40 (65.6%)	18 (18.8%)	7 (18.4%)	22 (39.3%)	23 (46.9%)	6 (42.9%)
Intracranial arterial stenosis stenting	42 (26.8%)	29 (47.5%)	13 (13.5%)	5 (13.2%)	13 (23.2%)	17 (34.7%)	7 (50.0%)
Extracranial arterial stenosis stenting	43 (27.4%)	34 (55.7%)	9 (9.4%)	4 (10.5%)	18 (32.1%)	16 (32.7%)	5 (35.7%)
Intracranial balloon dilatation	25 (15.9%)	19 (31.1%)	6 (6.3%)	2 (5.3%)	10 (17.9%)	8 (16.3%)	5 (35.7%)
Extracranial balloon dilatation	23 (14.6%)	16 (26.2%)	7 (7.3%)	3 (7.9%)	10 (17.9%)	6 (12.2%)	4 (28.6%)
Deep brain stimulation (DBS)	15 (9.6%)	7 (10.9%)	8 (8.3%)	1 (2.6%)	2 (3.6%)	7 (14.3%)	5 (35.7%)
Transcranial magnetic stimulation (TMS)	12 (7.6%)	6 (9.8%)	6 (6.3%)	1 (2.6%)	1 (1.8%)	6 (12.2%)	4 (28.6%)
Botulinum toxin therapy	31 (19.7%)	18 (29.5%)	13 (13.5%)	4 (10.5%)	11 (19.6%)	12 (24.5%)	4 (28.6%)
Muscle biopsy	23 (14.6%)	15 (24.6%)	8 (8.3%)	2 (5.3%)	7 (12.5%)	10 (20.4%)	4 (28.6%)
Electroencephalogram	129 (82.2%)	51 (83.6%)	78 (81.3%)	23 (60.5%)	49 (87.5%)	43 (87.8%)	14 (100%)
Electromyography	103 (65.6%)	48 (78.7%)	55 (57.3%)	11 (28.9%)	44 (78.6%)	36 (73.5%)	12 (85.7%)
Transcranial doppler	88 (56.1%)	45 (73.8%)	43 (44.8%)	11 (28.9%)	38 (67.9%)	27 (55.1%)	12 (85.7%)
Polysomnography (PSG)	76 (48.4%)	38 (62.3%)	38 (39.6%)	10 (26.3%)	30 (53.6%)	28 (57.1%)	8 (57.1%)
Drug metabolism genetic testing	41 (26.1%)	29 (47.5%)	12 (12.5%)	2 (5.3%)	16 (28.6%)	18 (36.3%)	5 (35.7%)
Drug concentration testing	88 (56.1%)	44 (72.1%)	44 (45.8%)	11 (28.9%)	39 (69.6%)	32 (65.3%)	6 (42.9%)
Oligoclonal bands testing	50 (31.8%)	29 (47.5%)	21 (21.9%)	6 (15.8%)	23 (41.1%)	17 (34.7%)	4 (28.6%)

Values are number (percent) when appropriate

^a^ The number of hospitals that have doctors who owns doctorate in neurology department

## Discussion

We found that a high rate of hospitals have neurology departments (83.1%) while less than half of the hospitals have stroke centers (41.0%) and neurology emergency services (23.6%). In available neurology departments, most of them can carry out intravenous thrombolysis (84.1%) while less of them have the ability to carry out arterial thrombectomy (36.9%). Our results indicate that stroke emergency services need to be enhanced in the county-level hospitals.

Acute neurological disorders, such as stroke, are associated with high mortality and disability and are a huge public health burden. Early management is associated with better outcome. In a previous study, 8–15% of patients admitted to emergency departments required neurological assessment [7, 8]. Our survey found that about a quarter of hospitals provide neurology emergency services, which indicates a large services deficiency and contributes to a serious problem. However, this situation is common in other countries, even in developed countries [9–11]. In our study, we found about 46.2% of hospitals can carry out multimodel CTA in emergency room. The advances and availability of neuroimaging will not only benefit the diagnosis but also become an important part of treatment guidance [12].

Among all acute neurological disorders, stroke is one of the most urgent events that requires prompt management [13]. In 1996, the US Food and Drug Administration approved intravenous tissue plasminogen activator (tPA) administered within 3 h of symptom onset as treatment for acute ischemic stroke [14]. However, less than 4.1% of ischemic stroke patients received this treatment [15–17]. Therefore, in 2000, recommendations for primary stroke centers were published in order to address the shortcomings in the infrastructure and organizations in hospitals [18]. Over the past 20 years, stroke units have become the standard for in-hospital stroke care [19]. However, clinical outcomes after stroke were reported substantially poorer in low-income and middle-income countries than in high-income countries [20]. Evidence-based treatments, diagnostics, and stroke units were less commonly available or used in low and middle-income countries [21]. In our study, we found that only 41.0% of county-level hospitals have stroke centers. Nevertheless, it is encouraging that in another 32.8% of hospitals, stroke centers are under construction, which will undoubtedly improve health care delivery for ischemic stroke patients. The findings that about 84.1% of the hospitals can carry out intravenous thrombolysis while 36.9% of hospitals have the ability to perform arterial thrombectory in county-level hospitals, pointed out several issues. Firstly, we should enhance the training to educate more neurologists and even internists so that more ischemic patients can receive treatment timely. Secondly, providing infrastructure and introducing expertise are also important. In China, medical education is classified as Bachelor of Medicine, Master’s degree of Medicine, and Doctoral degree of Medicine. We found only about 22.3% of hospitals have physicians who have doctorates. Finally, currently, not all hospitals have the ability to provide intravenous thrombolysis or even arterial thrombectory, it is crucial to build stroke units and enhance neurology network/stroke unit network so that stroke patients could be treated in time and if need be, transferred to the qualified hospitals (such as comprehensive a stroke center) for further effective treatments without delay. Currently, the time window of thrombolysis is increased from 3 to 4.5 h after acute ischemic stroke [22]. More recently, stroke thrombolysis was demonstrated to be safe and efficacious in a later treatment window (> 4·5 h from stroke onset) with appropriate patient selection using neuroimaging [23, 24]. It underscores the importance of extending the availability of stroke care resources and infrastructure in order to meet the clinical needs of the population with optimal treatment, reducing post-stroke burden.

Few previous studies have been performed regarding the structure and organizations of neurology departments in a wide range of county-level hospital facilities in China. Other available neurological care services are also shown in Table 2, providing information about the current condition in county-level hospitals. Availability of services for neurological disorders including stroke care in the study hospitals was varied. Overall, the tertiary hospitals are much better equipped with modern medical resources including neurology specialists, available procedures, interventions or treatments, when compared with the secondary hospitals. However, the secondary hospitals are more likely to receive the acute onset patients before referring to tertiary hospitals. According to the four administrative regions, the available neurological care services are aligned with economic levels. Compared with the other three other administrative regions, Anhui province ranked last economically (http://data.stats.gov.cn/english/). Accordingly, available neurological services in Anhui province are less equipped in almost all aspects. For examples, the hospitals in Shanghai are much equipped and standardized in practice than those hospitals in Anhui Province.

Our study has several limitations. First, it is not a national survey so it cannot represent the current hospital and neurology department conditions of the entire country of China, and it actually represents a relatively advanced health care area within China. Second, since not all county level hospitals in Yangtze River Delta Urban Agglomerations have neurology department, and not all hospitals we invited participated in our study, it may lead to the selection and reporting bias. Third, we did not include telemedicine networks as part of survey, which also serve as an important part of neurological services. Furthermore, our study mainly investigates hospital settings and department resources while lacking more detailed information such as assessments of the quality of diagnosis and treatment in each hospital. For example, we did not investigate the care timelines in stroke care due to resource constraints of the study, which needs to be included in further studies since monitoring care timelines is important for quality control of stroke care services.

## Conclusions

Our study found a high rate of hospitals with neurology departments while less than half of the hospitals have a stroke center and neurology emergency services. Tertiary are hospitals are much more equipped with modern medical resources compared with secondary hospitals. Our results indicate that stroke care services need to be improved at the county-level hospitals and which should improve health care delivery for the stroke patients to receive the best therapy.

## Data Availability

The data that support the findings of this study are available from supplementary material.

## Abbreviations

CTA: Computed tomography angiography
DSA: Digital subtraction angiography
tPA: Tissue plasminogen activator
